# Supplementary material for: D4Z4 Hypomethylation in Human Germ Cells
Source: Cells. 2024 Sep 6;13(17):1497. doi: 10.3390/cells13171497 (PMC11394335; doi:10.3390/cells13171497)
Supplement: Supplementary file 1 [file cells-13-01497-s001.zip › cells-3139848-supplementary.pdf]

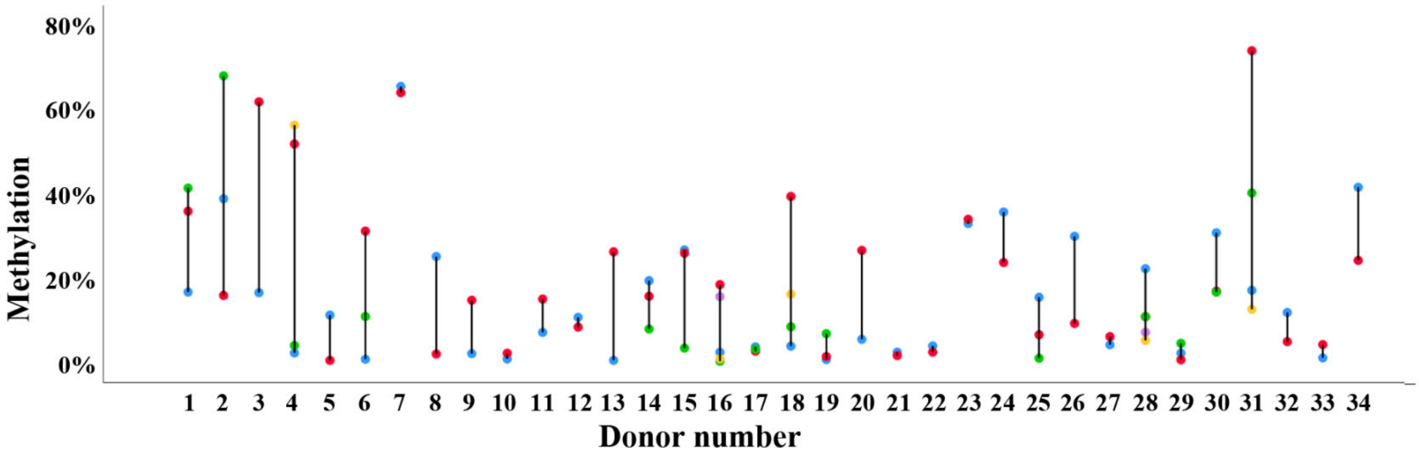

Figure S1. D4Z4 methylation variation in oocytes from the same donors. Different oocytes from a given donor (numbers 1-34) are indicated by differently colored (blue, red, green, yellow and mauve) dots on a vertical line.

Table S1. Semen parameters.

	Mean<br>± standard deviation	Range
**Donor Age (years)**	38.2±5.7	26.3-54.0
**Donor BMI (kg/m²)**	26.1±4.2	17.5-51.7
**Sperm volume (ml)**	3.2±1.3	0.2-8.0
**Sperm concentration (x 10⁶/ml)**	68.4±46.7	5.3-250
**Morphology (% normal)**	8.4±3.6	3.0-18.0
**Motility (% motile)**	53.7±11.8	35.0-85.0

Table S2. Clinical parameters of oocyte donors.

	Mean<br>± standard deviation	Range
**Oocyte donors (N)**	53	
**Donor age (years)**	34.3±3.8	28-42.7
**Donor BMI (kg/m^2)**	26.6±5.8	18.9-42.5
**Previous number of OPUs**	2.2±2.0	0-12
**OPUs for this study (N)**	56	
**Oocytes per OPU**	9.6±4.4	2-21
**GV oocytes per OPU**	1.9±1.0	1-5
**Day of embryo transfer**	3.9±1.0	3-6
**Number of embryos transferred**	1.7±0.5	1-3
**Clinical pregnancy (N)**	14	
**% Clinical pregnancy / OPU**	25	95% CI 13.7-36.3
**Group 1:<br>OPU with at least one GV oocyte<br>< 2.5% D4Z4 methylation (N)**	17	
**Clinical pregnancy (N)**	6	
**% Clinical pregnancy / OPU**	35.3	95% CI 12.6-58.1
**Group 2:<br>OPU with no GV oocyte<br>< 2.5% D4Z4 methylation (N)**	39	
**Clinical pregnancy (N)**	8	
**% Clinical pregnancy / OPU**	20.5	95% CI 7.8-33.1

OPU = Oocyte pick-up; BMI = Body mass index; GV = Germinal vesicle; 95%CI = 95% confidence interval

Table S3. Primers for multiplex (outer) and singleplex (inner) PCR and pyrosequencing.

Target region	Primer	Sequence (5′-3′)[a]	Number of CpGs	Annealing temperature
DUX4	Forward outer	AGGAAGGTAGGGAGGAAAA		59°C
	Reverse outer	ACAAAACTCAACCTAAAAATATAC		
	Forward inner	GGGTTGAGGGTTGGGTTTATA		58°C
	Reverse inner	*ACAAAACTCAACCTAAAAATATAC		
	Sequencing	GGGTTGGGTTTATAGT	9	
GTL2 (paternally imprinted)	Forward outer	GAATTATAGGGAATGATGGTGTA		59°C
	Reverse outer	CCAAAATACTAACTACTCCTTAAACA		
	Forward inner	AGGGTTAGGAAGTTTAGTAGGTTA		60°C
	Reverse inner	*ACTACTCCTTAAACAAAAAAACACATAAT		
	Sequencing	GTAGTAAATTAAAGTGTATTAGAGA	2	
PEG3 (maternally imprinted)	Forward outer	GGTTGTTGATTGGTTAGTATAG		59°C
	Reverse outer	CACTCACCTCACCTCAATAC		
	Forward inner	GGTGTAGAAGTTTGGGTAGTTG		57°C
	Reverse inner	*CTCACCTCACCTCAATACTAC		
	Sequencing	TGTTTATTTTGGGTTGGT	4	

[a] Primers indicated by a star are biotinylated at the 5' end.

Table S4. Primers for deep bisulfite sequencing.

Assay	Primer	Sequence (5'-3')[a]	Amplicon length	Number of CpGs	Annealing temperature
DUX4	Forward	GAAGGTAGGGAGGAAAAG	259 bp	30	58°C
	Reverse	ACAAAACTCAACCTAAAAATATA			
